# Supervision for Aspiring Behaviour Analysts in Australia: An Exploration of Current Practices, Challenges, and Opportunities

**DOI:** 10.1007/s40617-022-00739-z

**Published:** 2022-09-26

**Authors:** Kristin Bayley, David Trembath, Erin Leif

**Affiliations:** 1 Griffith University, Southport, Australia; 2 Monash University, Clayton, Australia

**Keywords:** Behavior analysis, Certification, Supervision, Fieldwork experience, Mentoring, Telesupervision

## Abstract

Effective supervision is a key component of the development of effective and ethical behavior analytic repertoires. However, the provision of supervision may be challenging in countries where behavior analysis is an emerging profession and there are few qualified practitioners. We conducted a mixed-methods survey study to examine the supervision practices of board certified behavior analysts (BCBAs) in Australia, and perceived challenges related to the provision of supervision. Respondents reported using a variety of supervisory practices to meet the demand for supervision, but a lack of time, resources, and geographical location posed challenges. Based on these findings, we provide several recommendations for addressing identified challenges. Although each recommendation has been contextualized to meet the needs of the Australian behavior analytic community, these recommendations may be useful in other parts of the world where behavior analysis is an emerging profession or there are few BCBAs to meet the needs of a growing behavior analytic workforce.

## Introduction

Over the past decade, opportunities and government support for the professional practice of applied behavior analysis (ABA) in Australia have increased in the education sector (see Department of Education & Training, 2022), the disability sector (see National Disability Insurance Scheme, 2020a; NDIS Quality & Safeguards Commission, 2019) and in early years support sector for children with autism and related conditions (see Prior et al., 2011). This has seen a commensurate increase in demand for services bolstered by government funding for supports in the sector at large (National Disability Insurance Scheme, 2020b), the establishment of a national ABA association (Association for Behaviour Analysis Australia, n.d.), the recent creation of two verified university training programs (Association for Behavior Analysis International, n.d.), and a marked increase in the number of board certified behavior analysts (BCBAs) and Board Certified Assistant Behavior Analysts (BCaBAs) in Australia (Behavior Analyst Certification Board, 2020a). This has been accompanied by a significant increase in the number of students enrolled in verified university training programs in Australia. Although promising, these developments highlight the need for effective and ethical supervision for Australian students of behavior analysis (i.e., trainees) as a matter of priority. At present, little is known about the capacity of the current Australian behavior analytic workforce to meet this need, or the common models of supervision used by Australian BCBAs who deliver supervision to trainees as part of their role.

### Benefits of Supervision

Supervision has purported benefits for trainees, the direct recipients of ABA-based services, and the profession of behavior analysis. First, supervision assists the trainee to develop new skill sets. The supervisor is responsible for oversight of the conceptualization, implementation, and evaluation of the behavior analytic services delivered by the trainee, including ethical decision making (LeBlanc & Luiselli, 2016; Sellers et al., 2016a; Valentino et al., 2016). As part of structured supervision, new skill sets on part of the trainee may be developed and refined through the use of evidence-based supervisory practices, including goal setting, task clarification modeling of new skills, rehearsal, and performance feedback (Garza et al., 2018; Parsons et al., 2012;  Sellers et al., 2016b; Reid et al., 2012). Second, supervision is likely to benefit the direct recipients of ABA-based services. Dixon et al. (2016) analyzed a large archival database of children receiving community-based ABA services to assess the impact of supervision intensity, supervisor credential, and caseload size on outcomes for children (defined as mastery of individualized learning objectives). A significant correlation was found between the credentials held by the supervisor and mastery of learning objectives, with children receiving supervision from a BCBA mastering 73.7% more learning objectives than children who received supervision from a professional without the BCBA credential. Finally, the provision of high-quality supervision directly benefits the profession of behavior analysis, in that it facilitates the delivery of high quality to services to clients and contributes to the professional development of both the supervisee and supervisor (LeBlanc & Luiselli, 2016).

### Challenges Associated with Supervision

Although successfully completing a period of supervised independent fieldwork is a requirement for trainees, accessing supervision in Australia may be difficult. Many trainees do not have local or “in house” access to a BCBA or qualified supervisor who is approved to deliver supervision as part of the conditions of their employment. Thus, trainees may need to seek external supervision from a qualified supervisor who is not affiliated with their employer. This requires trainees to identify an approved supervisor in their local community or, in many cases, in different parts of the country or world. Entering a supervisory relationship with an external supervisor may present challenges. First, the trainee must ensure that they are able to undertake approved behavior analytic activities that meet the requirements of the Behavior Analyst Certification Board (BACB), as part of their professional work. Second, the trainee must ensure that the supervisor is knowledgeable about the local policies, procedures, and cultural considerations that influence and inform their professional work. Third, the trainee and supervisor must ensure that consent is obtained from clients to share client-specific information and that client-specific information is shared in a way that protects client privacy and confidentiality, in accordance with local laws. Finally, the use of videoconferencing to deliver remote supervision may limit the degree to which the trainee and supervisor can incorporate modelling and rehearsal of new skills into direct observations and supervision meetings. This may also make it difficult for the supervisor to support the trainee during high-risk situations, such as effectively and safely responding to behaviors of concern (Turner et al., 2016).

Trainees who seek external supervision typically enter a fee-for-service relationship with the supervisor. Supervisors typically charge an hourly rate for such services, which is determined by the supervisor. Because trainees pay for services, supervisors and trainees must also consider the potential negative impact that the fee-for-service arrangement may have on the professional relationship (Turner et al., 2016). In addition, the cost of supervision may be prohibitive for some trainees, preventing them from accessing high quality supervision from experienced supervisors. At present, there is no regulated fee structure for supervision, and thus costs may vary considerably depending on format, location and demand.

Finally, the provision of effective and ethical supervision to trainees requires time and resources on the part of the supervisor (Sellers et al., 2016a). Per the BACBs *Board Certified Behavior Analyst Handbook* (BACB, 2020b), the supervisor must directly supervise a minimum of 5% of the fieldwork hours accrued by the trainee each month. If a trainee accumulates 30 hr of fieldwork per week, the supervisor must deliver three hours of supervision per fortnight. However, Turner et al. (2016) described the additional time that must often be allocated by the supervisor above and beyond the hours of direct supervision, including (1) travel time to and from the trainee’s place of work (to conduct direct observations, if video conferencing is not an option); (2) travel time to and from supervision meetings; (3) time to prepare for supervision, which may include reviewing written documents prepared by the trainee and identifying and reviewing relevant literature related to client programming; and (4) time to engage in other supplemental work, such as responding to trainee emails, completing trainee performance evaluations, documenting supervision, and engaging in professional development related to the provision of effective and ethical supervision. It is likely that the actual time required to deliver effective supervision to trainees is double the number of hours associated with the direct delivery of supervision (e.g., observations and meetings; Sellerset al., 2016a).

### Supervision in the Australian Context

At present, the only regulatory framework for ABA professionals in Australia is the North American-based BACB. In 2019, the BACB Board of Directors announced that the BACB would substantially revise its international focus and would cease accepting certification applications from trainees residing in countries outside North America from 2023 (BACB, 2019). Following a period of deliberation with the BACB, Australia was granted an exemption, and, for the foreseeable future, Australian trainees who meet specific coursework and supervised fieldwork requirements (BACB, 2020b) will be able to apply for BACB certification. Per the BACBs fieldwork requirements, Australian trainees who wish to sit for the professional certification exam will need to accrue 2,000 hours of supervised independent fieldwork or 1,500 hours of concentrated fieldwork, in which 5% (10% for concentrated fieldwork) of the total hours of fieldwork each supervisory period (1 calendar month) is directly supervised by a qualified supervisor (BACB, 2020b). Thus, the provision of supervision that meets BACB fieldwork requirements will continue to be an important area of need for the foreseeable future, as the profession of ABA develops in Australia.

In 2020, Leif et al. conducted the first national survey of Australian ABA practitioners to identify (1) the education and training experiences of ABA practitioners; and (2) the barriers ABA practitioners faced when designing, supervising, and delivering ABA-based programs in Australia. From the survey of 152 program supervisors, 80% reported having a graduate degree, but only 31% reported holding the BCBA credential. These findings may reflect the fact that some practitioners in Australia have difficulty accessing supervision to meet the eligibility requirements to sit for the professional certification exam, and/or that there are not currently enough BCBAs to meet the demand for ABA-based services. In addition, the findings of the survey highlighted specific challenges related to the provision of supervision. The most common barriers identified by survey respondents were access to high quality and cost-effective training, supervision, and professional development. Practitioners indicated that their current supervision and professional training opportunities were inadequate, and they highlighted a need for high-quality training programs, qualified supervisors in the field, more supervision hours and more professional development opportunities such as conferences, workshops, and webinars. The need for access to supervision as a matter of priority was evident especially considering that the majority of survey respondents had less than 5 years of experience working in the field, with many reporting only 1–2 years of professional experience.

### Purpose

Given the recent increase in demand for behavior analytic services in Australia and the recent establishment of two verified university course sequences in ABA, it is anticipated that the demand for supervision will continue to grow. To date, no research has explored the provision of supervision to trainees in Australia. However, there is a need to identify how many BCBAs in Australia are currently providing supervision, how these supervision experiences are structured, and whether these supervisors have the capacity to increase the amount of supervision they provide as demand continues to grow. Therefore, the purpose of the current study was to survey BCBAs in Australia to determine their current and future capacity to deliver supervision to trainees. To this end, we aimed to identify the proportion of BCBAs who currently deliver supervision as part of their role, and the number of trainees they currently supervise, and their estimated capacity to supervise new or additional trainees in the future. A second aim of the study was to identify and describe the models that Australian BCBAs currently use to deliver supervision to trainees, including mode (e.g., in-house versus external; in-person vs. telesupervision) and format (e.g., individual vs. group supervision). A third aim of the current study was to identify the barriers that Australian BCBAs face when delivering supervision to trainees, and to propose potential solutions to commonly identified barriers.

## Method

### Design

We used a custom online mixed-methods survey to examine the supervision practices of BACB certificants (BCaBAs, BCBAs, and BCBA-Ds) in Australia.

### Participants and Recruitment

Participants were recruited for this study via email and social media. The first and third author sent email invitations to complete the survey to Australian professionals with a BCBA qualification (i.e., professional colleagues) and to Australian organizations that employ BCBAs. Snowball sampling was used, in which respondents were invited to share the survey link with other potential respondents. Emails contained a brief summary of the purpose and aims of the study, contact details of the authors, and a link to access the survey on REDCap. In addition, a link to the survey was shared by the first and third author on LinkedIn, and to two Australian ABA Facebook pages. Potential respondents were invited to click a link to access the electronic survey at a time and place of their choice. They were first asked to read a short explanatory statement describing the aims of the survey, the potential benefits and risks of participating, the voluntary nature of participation, and how to contact the research team with questions. To be eligible to participate in the survey, respondents were required to hold an active professional credential as BCaBA, BCBA, or BCBA-D. BACB certificants were invited to participate if they were or were not currently delivering supervision to trainees. Allied health professionals or professionals with a master’s degree in ABA who supervised ABA-based programs in Australia but who did not hold a BACB credential were excluded from participating. Finally, respondents were asked to electronically provide their consent to participate. Once consent was provided, respondents were able to view and answer the survey questions.

Data were retained and analyzed for 39 participants. At the time that the survey closed (August 2020) there were 149 BCBA, BCaBA, and BCBA-Doctoral certificants listed on the BACB certificant registry in Australia, meaning that 26.2% of eligible Australian certificants completed the survey. Table 1 depicts the demographic characteristics of participants. Most respondents were BCBAs (90%), with the remaining 10% holding a BCaBA or BCBA-Doctoral credential. Approximately half of the respondents were dually certified as behavior analysts and allied health professionals (e.g., psychologists or speech pathologists, 17.5%) or registered teachers (35%). Most respondents worked in private practice, with the number of people employed within their workplace varying from 1 (e.g., sole traders) to over 100. Most respondents were employed full time, and 27.5% of respondents were the business owner. Respondents resided primarily in New South Wales (39.4%), Victoria (21.2%), South Australia (18.2%), and Queensland (15.2%). The majority of respondents obtained their certification between 2014 and 2020 (72.5%), and most respondents reported having worked full time for fewer than 5 years since becoming certified (75%).

**Table 1:** Participant demographics

Demographic Category	Demographic Details	*n* (%)
Certification Level	BCaBA	2 (5%)
	BCBA	36 (90%)
	BCBA-D	2 (5%)
Other Professional Qualifications	Allied Health (Psychology. Speech, Occupational Therapy)	7 (17.5%)
	Education	14 (35%)
	Other	3 (7.5%)
Employment Type	Private Practice	29 (72.5%)
	Not-for-Profit	8 (20%)
	Education Sector	5 (12.5%)
Employer Size	1 employee (sole trader)	4 (10%)
	2–5 employees	5 (12.5%)
	6–10 employees	3 (7.5%)
	11–20 employees	5 (12.5%)
	21–50 employees	8 (20%)
	51–100 employees	6 (15%)
	100+ employees	10 (25%)
Employment Status	Business Owner	11 (27.5%)
	Employee	29 (72.5%)
Employment Fraction	Full Time	32 (80%)
	Part Time	8 (20%)
State or Territory	Australian Capital Territory	1 (3%)
	New South Wales	13 (39.4%)
	Northern Territory	0
	Victoria	7 (21.2%)
	Queensland	5 (15.2%)
	South Australia	6 (18.2%)
	Western Australia	1 (3%)
	Tasmania	0
Years certified with the BACB	2006 and earlier	1 (2.5%)
	2007	1 (2.5%)
	2009	1 (2.5%)
	2011	2 (5%)
	2012	2 (5%)
	2014	4 (10%)
	2015	3 (7.5%)
	2016	6 (15%)
	2017	5 (12.5%)
	2018	6 (15%)
	2019	5 (12.5%)
	2020	4 (10%)

### Survey Development and Distribution

A custom online survey comprising 36 questions and distributed via REDCap was developed by the authors to address the research aims (see Appendix 1). Part 1 focused on demographic information including participants’ professional qualifications, experience, and workplace. Part 2 focused on participants’ experiences providing supervision to those pursuing BCaBA/BCBA certification including mode of supervision (in person or telesupervision), current and future anticipated supervision loads, organizational context in which supervision was provided, supervision format (individual or group), and fees. Data were collected using a mix of Likert-type, closed-ended, and open-response questions. Data were collected between May and August 2020.

### Data Analysis

Survey data were exported from REDCap to SPSS (version 27) for analysis. Descriptive statistics were used to summarize quantitative data in relation to the study aims. Where exploratory analyses were conducted to further elucidate the findings, nonparametric tests were used (Mann-Whitney U Test, Spearman’s correlations) due to the small sample size and use of Likert scale interval data. Interpretation of these tests focuses on effect sizes (eta squared, correlation coefficient) as opposed to *p*-values, which are reported for completeness only, consistent with contemporary recommendations (e.g., Staggs, 2019).

Responses to open questions regarding perceived barriers and strategies related to supervision were analyzed using content analysis based on the principles outlined by Dey (1993). The following steps were completed: (1) the first and second author reviewed all responses and independently drafted a set of codes and operational definitions to account for the data; (2) the first and second author met to review and refine the codes until consensus was achieved; (3) the second and third author independently applied the coding scheme to the data; and (4) the second and third author compared the coding, calculated reliability, and resolved any disagreement by consensus. The first author was available to resolve any disagreements, but this was not required. Cohen’s Kappa was used to calculate agreement between the second and third author for coding related to the three questions asked of participants: barriers for supervisors (*k* = 0.801, *p* < .001), barriers for supervisees in accessing supervision (*k* = .748, *p* < .001), and proposed strategies to support supervision (*k* = .657, *p* < .001) reflecting moderate to strong agreement (Landis & Koch, 1977).

### Data Screening

The survey was accessed 41 times, with sufficient data to determine eligibility available for 40 respondents. One additional respondent was excluded because they indicated they did not have a relevant behavior qualification (BCaBA, BCBA, BCBA-D). Optional potentially identifying demographic information was not provided by one participant. Data were missing for three questions regarding the provision of in-person (q22, 2 missing) and/or telehealth (q24, 3 missing), and whether the cost of supervision is likely to rise (q31) but in each case could be explained by prior questions (e.g., these respondents did not offer supervision). One participant did not respond to the question regarding future supervision capacity (q43).

## Results

### Offering of Supervision

**Fig. 1:**
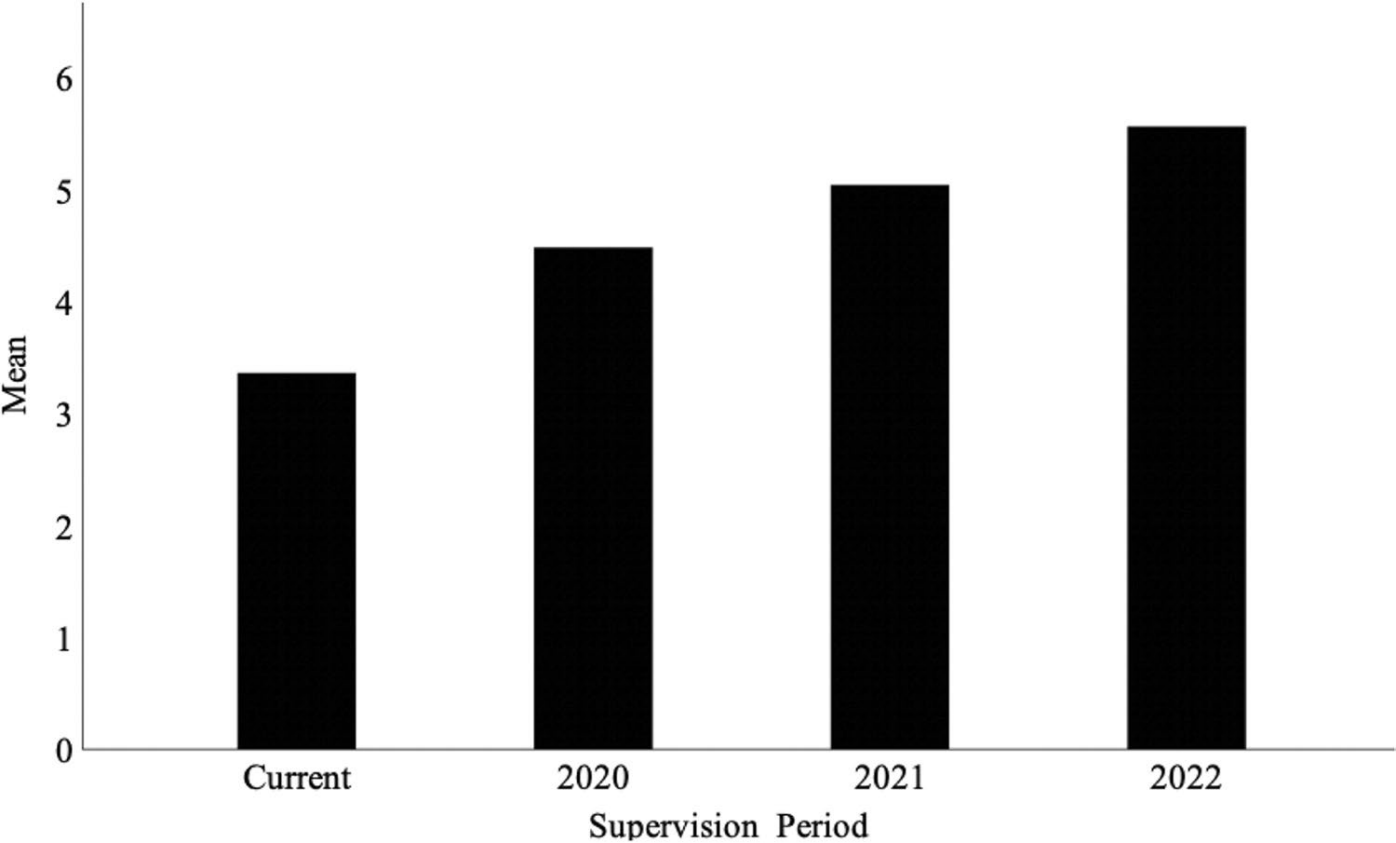
Current and anticipated supervision load for the period 2020–2022

**Fig. 2:**
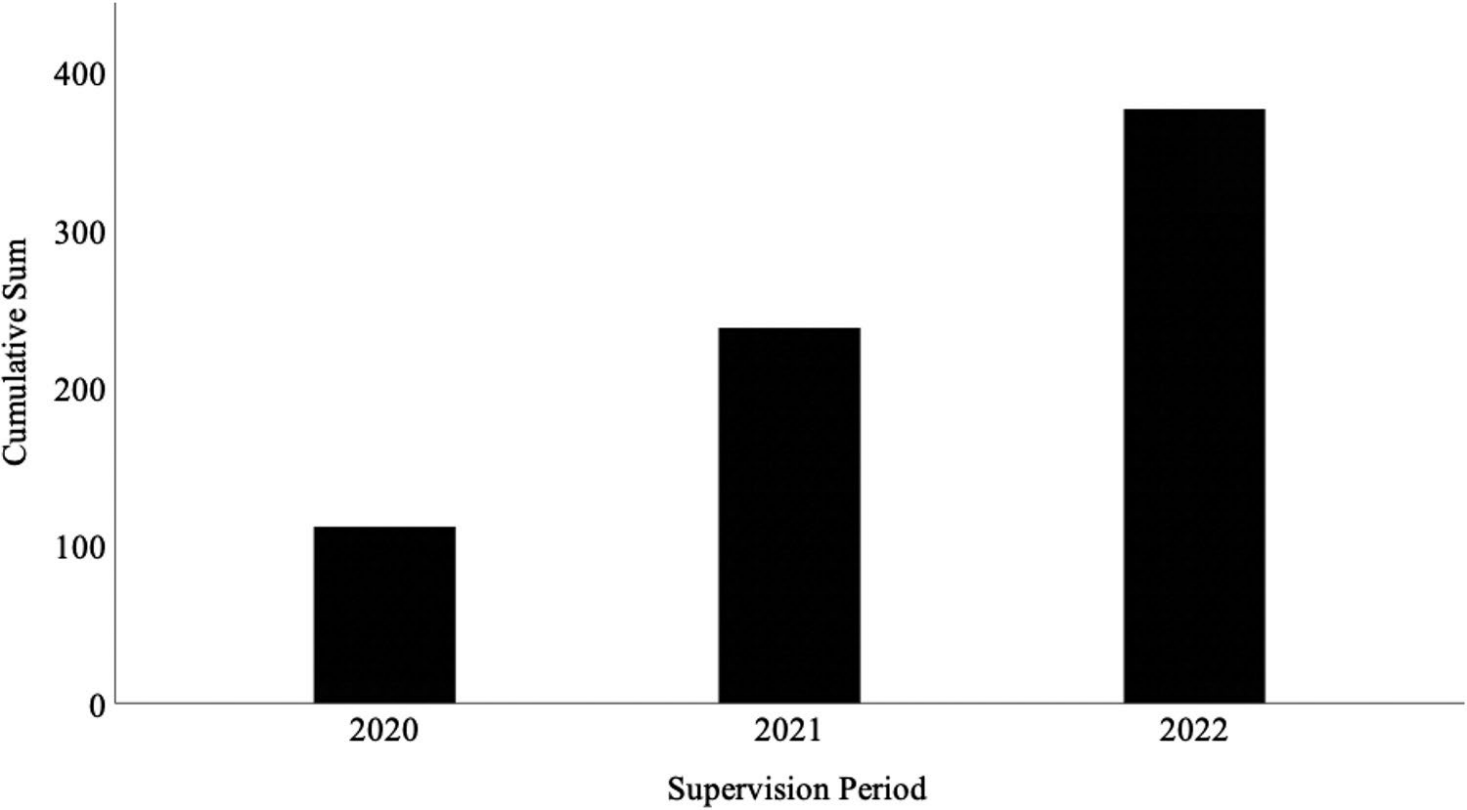
Cumulative count of anticipated supervision offerings over the period 2020–2022

The first aim of the study was to identify the proportion of suitably credentialled respondents who had provided supervision, or anticipated doing so, in the ensuing 3-year period (2020–2022). Twenty-nine of the 39 respondents indicated that they had previously provided supervision, with a mean of 7.28 (*SD* = 7.44, range: 1–30) supervisees. This included 26 of 36 BCBAs and the two BCBA-Ds. At the time of the survey, 29 respondents (27 BCBAs, 2 BCBA-D) were offering supervision and 36 (including 1 of 2 BCaBAs) indicated that they intended to offer supervision in the next 3 years. As illustrated in Fig. 1, there was evidence of a positive trend in the number of supervisees respondents anticipated supervising in the ensuing 3-year period, increasing from the currently reported 3.93 (*SD* = 4.11, range: 0–20) to the anticipated mean of 4.79 (*SD* = 4.983, range: 0–20) in 2022. Note that the survey was completed mid-2020, hence the difference between the number identified as current and the anticipated total for 2020. Figure 2 illustrates the cumulative effect of supervision on workforce capacity over the same period.

### Exploratory Analysis of Factors that May Influence Supervision Practices

We completed exploratory analyses to examine the possible association between demographic characteristics (years of professional experience, prior supervision experience) and current supervision practices. A Mann-Whitney-U Test revealed that as a group, respondents who were currently offering supervision had more years of professional experience (*n* = 29, Mdn = 3, range: 1–4) than those who were not (*n* = 11, Mdn = 2, range: 1–5) with a small effect size, *U* = 91, z = -2.143, *p* = .032, *n*^*2*^ *=* 0.118. There was a positive correlation between the respondents’ years of experience and the number of people they were currently supervising to become BCBAs, *r* = 0.475, *p* = .005. Twenty-four of the 40 participants were providing supervision currently and had done so in the past, 5 were supervising currently but had not done so in the past, 5 had done so in the past but were not currently, and 6 participants had not offered supervision.

We completed similar analyses of these factors in relation to the respondents’ plans for future supervision. There was again a small difference in the years of professional experience between groups, with those indicating that they would offer supervision in the ensuing period having marginally more experience (Mdn = 3, range: 1–5) than those who did not anticipate offering supervision (Mdn = 2, range: 1–4), with a small effect size, *U* = 59.50, z = -.582, *p* = .560, *n*^*2*^ *=* 0.014. There were positive but progressively weakening correlations between the respondents’ years of experience and the number of people they were intending to supervise in 2020 through to 2022 (*r* = .535, .389, .277, respectively). A total of 10 respondents indicated that they would provide supervision for the first time in the ensuing 3-year period, whereas two respondents who had previously provided supervision did not intend to do so in the same period.

### Supervision Delivery

The second aim of the study was to identify and describe the models that Australian BCBA certificants currently use to deliver supervision to trainees.

#### Supervision Context

**Fig. 3:**
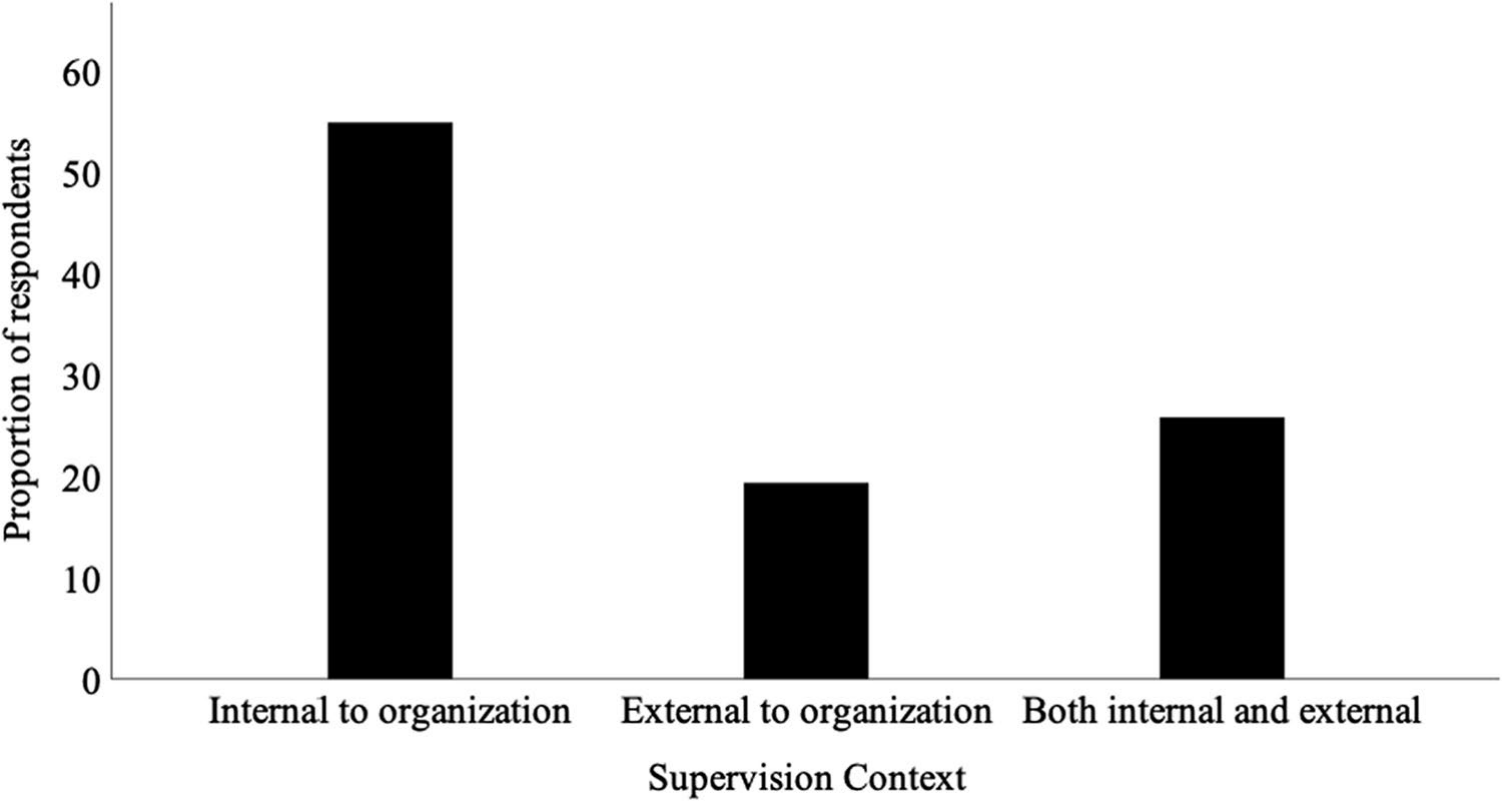
Organisation context in which supervision was provided

Figure 3 presents the proportion of respondents providing supervision in each of the organizational contexts. Of respondents who had offered or were currently offering supervision, 17 had provided supervision to supervisees within their organization only, 6 to supervisees external to their organizations only, and 8 to supervisees within and external to their organization.

#### Delivery Mode and Format

**Fig. 4:**
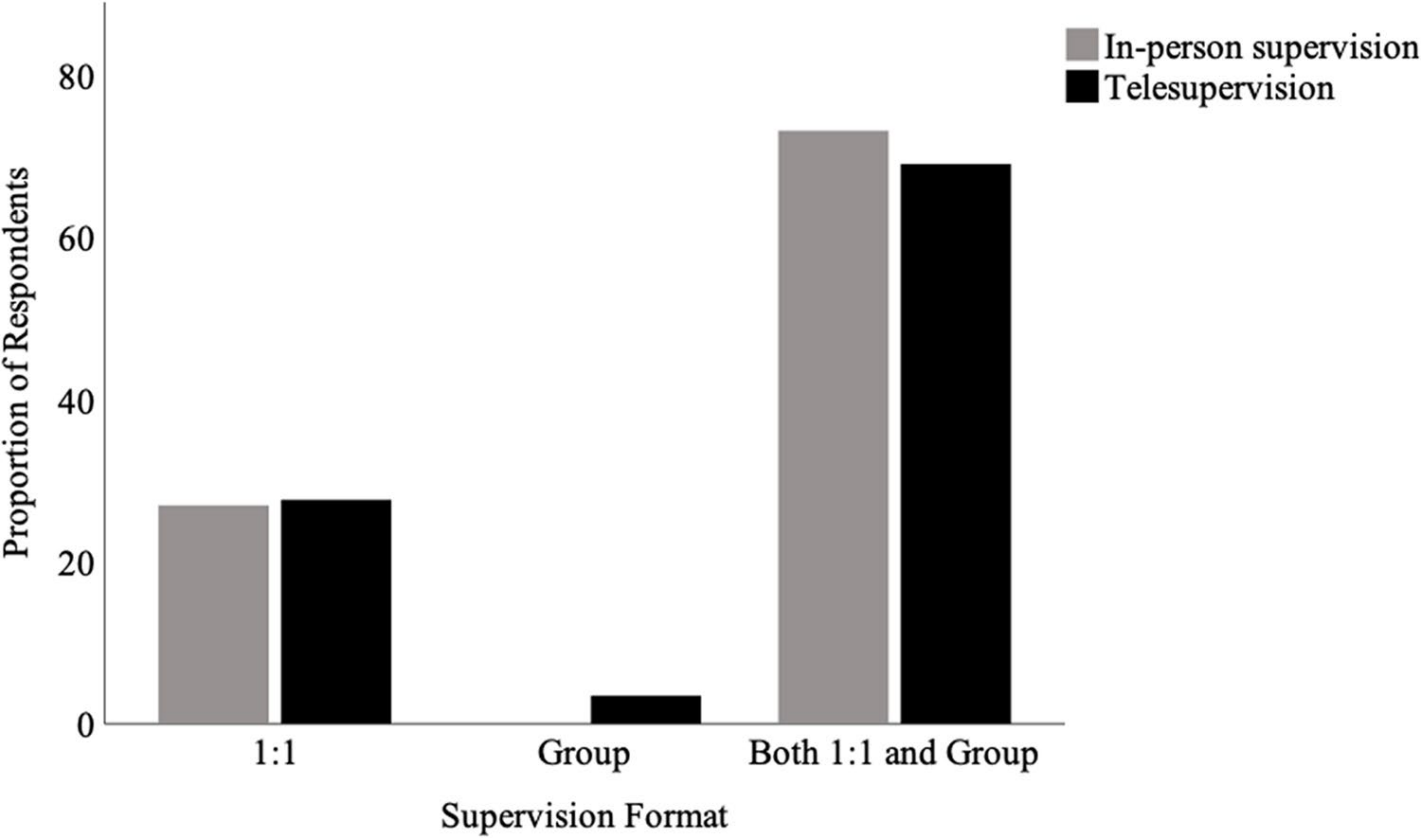
Supervision format for in-person and telesupervision

Figure 4 summarizes the format (1:1, group, both 1:1 and group) of supervision used in each of the two modes (in-person, telesupervision). Regarding the mode of supervision, 2 respondents (5.9%) provided supervision in-person only, 5 (14.7%) provided telesupervision only, and 24 (70.6%) provided both in-person and telesupervision. It should be noted that for each mode, there were negligible differences, with respondents almost always offering either 1:1 or a combination of 1:1 and group supervision.

#### Payment for Supervision

Respondents were asked to indicate yes/no in relation to each of four potential payment arrangements. In the most common arrangement people accessed supervision as part of their employment (70.59%), followed by direct payment (35.29%), payment to the supervisors’ organization (11.76%), and access as part of their employment but with an additional fee (2.91%). Sixteen respondents provided a monetary figure for the cost per/hour of supervision they provided, with a mean value of $100.47 AUD (*SD* = 44.14, range: 40–215). Two of the 16 respondents provided separate amounts for individual versus group supervision, which in both cases were $40 for individual and $25 for group. One participant (in addition to the 16 respondents) indicated that the amount charged is “depending on continent and current role.” Exploratory analysis revealed a small negative correlation between the amount charged and full-time equivalent years of experience as a BCBA certificant (*r = -2.56, p* = .338), and a moderate negative correlation between the amount charged and the number of people previously supervised (*r* = -.413, *p* = .183).

Seven respondents indicated that they would not increase the fee they charged within the next 24 months, and one was unsure. One participant indicated they would increase their fee in line with payment schedules for therapy delivery within the National Disability Insurance Scheme (NDIS), and two respondents indicated they would increase their fee but did not specify by how much. The remaining six respondents indicated they would increase their fees by 50%, 25%, 19%, 25%, and 25%, equating to an increase of between $10 and $50 per hour.

### Barriers and Solutions to Supervision in Australia

The third aim of the study was to identify perceived barriers and solutions related to the successful delivery of high-quality supervision in Australia into the future. Respondents were asked to rate their level of agreement on a 5-point Likert scale (strongly disagree, disagree, neutral, agree, strongly agree) to four statements regarding supervision capacity. The median response was “disagree” for statements “there are currently enough supervisors in Australia to meet student demand” (mode = “disagree,” range = “strongly disagree” to “agree”) and “In the next 3 years, there will be enough supervisors in Australia to meet future demand” (mode = “disagree,” range = “strongly disagree” to “neutral”). The response was more positive in relation to respondents indicating both knowledge and confidence to provide supervision (irrespective of whether they were currently doing so) with consistent findings (median = “agree,” mode = “agree”, range = “disagree” to “strongly agree”).

Table 2 depicts thematic categories and definitions of barriers to delivering supervision, barriers to accessing supervision, and potential solutions to barriers described by survey respondents in response to the three open survey questions. A range of barriers to the provision of supervision were identified. Of three respondents who indicated they would not be offering supervision in the ensuing 3-year period, the first cited the reason as “personal choice,” the second cited time constraints due to research and administration responsibilities, and the third cited time constraints and concerns about their ethical responsibility for supervisees’ caseloads in cases where supervisees were external to their own workplace. Content analysis of responses from the broader sample yielded 10 thematic categories relating to their perceived barriers for supervisors. The most commonly identified barriers for supervisors were related to a lack of time for supervision activities (time; 42%) and geographical location (location; 27%). Seven categories relating to their perceived barriers for people seeking supervision were identified, with the most common barriers related to a lack of available supervisors (supply; 54%), a lack of opportunities for supervisees to find and contact potential supervisors (networking opportunities; 26%), and challenges finding supervisors with the right knowledge, skills, and competence to match supervisee needs (supervisor competence; 21%). Potential solutions for addressing these barriers were organized into eight thematic categories. Respondents suggested that barriers associated with the provision of supervision could potentially be remediated by providing more supervisor training and support (31%), creating university-affiliated fieldwork placements (20%), and providing greater financial support for supervision activities (20%).

**Table 2:** Thematic categories and definitions of barriers to delivering supervision, barriers to accessing supervision, and potential solutions to barriers, the percent of statements related to each thematic category, and illustrative quotes from open-ended survey responses

	Theme	Definition	%	Illustrative Quotes
Barriers for Supervisors	Time	Constraints or barriers related to a lack of time to provide supervision, which may be expressed in terms of time, capacity, and/or competing demands that imply that a lack of time is the primary concern.	42%	Our organization is small and our caseloads are full. Taking on supervisees is important to grow our field but sacrificing time that could be spent with clients to supervise isn't a viable choice for our organization. Time pressures of those in a position to supervise. Very little time…given to supervisors to provide and prepare for adequate supervision. Time constraints (working full time and providing supervision).
	Location	Constraints or barriers related geographical location	27%	Travel distances. Geography.
	Support—Training and Certification	Constraints or barriers related to a lack of resources/materials/training opportunities/networking opportunities to support supervisors to obtain and maintain supervisory competency	18%	Uncertainty of BCBA being recognized as a profession and therefore willingness to invest further time/expense in maintaining certification. (Lack of) resources (supervision curriculum). Lack of resources that reflect culture and education system in Australia.
	Quality Control	Concerns related to one’s own capacity to ensure high quality service provision by the supervisee	15%	(There are a) large number of supervisees to relatively few experienced supervisors I don't like to supervise people outside of organization. This is due to the time it takes, the quality of supervision provided and the liability that rests on the supervisor. Ensuring quality practice within other agencies given the relative infancy of ABA within Australia.
	Supply	Constraints or barriers related to a lack of available supervisors	12%	Volume of supervisees requiring supervision needing to be balanced with number of families requiring services from a BCBA (i.e., cost/benefit of providing training to future BCBAs now to the cost of being able to provide fewer families support as a result of this) There are too few BCBAs, and we're needed for intervention.
	Scope of Practice	Constraints, barriers, or issues related to one’s own scope of practice in supervision	9%	Pressures to provide supervision that is outside of scope. Limitations on scope based on legislation/Government policy or client preferences.
	Personal Attributes	Constraints, barriers, or issues related to one’s own self-reported attributes including knowledge, skills, confidence, and experience	9%	(I’m) not highly skilled in providing supervision. (I’m) not feeling confident providing external and/or remote supervision.
	Support—Governing Bodies	Constraints or barriers related to oversight by a national or international body that is relevant to the work (including supervision) of behavior analysts	6%	(The) lack of a unified institution body to regulate and standardize the practices for supervisors and supervisees.
	Support—Employer	Constraints or barriers related to a lack of support provided by the supervisors’ employer	6%	Not enough support from employer.
	Financial	Constraints or barriers related to financial considerations related to delivering supervision, costs of obtaining supervision, and fees for supervision	6%	Funding sources.
	Supervisee Responsibly	Constraints or barriers related to supervisees (potential or actual) not fulfilling their responsibilities		. . . very limited understanding of people seeking supervision of the requirements—this is the biggest one. Supervisees seek out supervision but haven't done any independent research around the requirements, how many hours are needed, restricted vs. unrestricted etc.
Barriers for Supervisees	Supply	Constraints or barriers related to a lack of available supervisors	54%	Not enough experienced supervisors. Not enough experienced BCBAs to go around, or in our case (we are) not willing to take on supervisees not working for us. . . . many of our candidates still get remote supervision overseas because there are not enough supervisors to go around. Lack of supervisors, especially supervisors who are willing to provide external supervision and/or remote supervision.
	Networking Opportunities	Constraints or barriers related to a lack of opportunities to contact eligible supervisors	26%	From what I've heard it can be difficult to find supervision opportunities if you aren't already working in the field. Supervisees already working as (supervisees) can often get supervision through their workplace, but external supervision can be difficult to find.
	Supervisor Competency	Constraints or barriers related to a lack of knowledge, skills, and/or experience on the part of supervisors, which may be general in nature or related to a specific area or aspect of practice	21%	Have taken over supervision for people who had accumulated 750 hr but were unable to pass RBT exam. Did not know basic terminology. . . . volume of supervisors available with knowledge in the specific area of practice required. Finding a supervisor who has skills relevant to your learner group.
	Fieldwork Opportunities	Constraints or barriers related to a lack of available placement/practicum opportunities necessary to obtain certification	18%	Lack of suitable practicum sites. Supervisee not having enough hours (of fieldwork). (Lack of) appropriate workplaces to conduct supervision effectively.
	Financial	Constraints or barriers related to financial considerations related to costs of obtaining supervision and fees for supervision	18%	Cost. Required to work for a company that offers it pay for privately. lack of resources such as funding and understanding of the need for supervision when providing ABA services.
	Location	Constraints or barriers related geographical location	15%	Location/ proximity to supervisor. …zero supervisors within their region. Travel distances.
	Outside Organization	Constraints or barriers related that may arise from a supervisor and supervisee working in different organizations	10%	(Supervisees are) often not within the organization of the supervisor which makes it logistically difficult as well as gaining client consent for supervision purposes.
Proposed Solutions	Supervisor Training and Support	Strategies/resources/materials/training opportunities/networking opportunities that may support supervisors to obtain and maintain competency	31%	More practical courses for supervisors. Resources and training available in Australia to develop supervisory skills. Mentorship programs for newer BCBAs to receive ongoing supervision so that they can become effective supervisors. Clear guidelines of the responsibility of the supervisor if providing external supervision (i.e., responsibility for the client receiving ABA services from the supervisee).
	University Placements	The provision of supervised experience via university run clinical placements/practicums/fieldwork	20%	Have the universities establish relationships with organizations as designated supervision sites. University student placement program. Universities starting placement programs with ABA services attached to universities.
	Financial Support	The provision of financial support to help supervisors and/or supervisees engage in the process	20%	Better funding for behavior analytic services to allow service providers to invest in supervision roles without putting financial strain on the company.
	Supervisor Supply	Increasing the number of available supervisors	14%	Proactive hiring of supervisors within government departments to support those completing coursework requirements, to allow them to complete fieldwork in a timely manner.
	Flexible Delivery	Using telesupervision or making other changes to supervision delivery that may make it more accessible to supervisees	11%	Telesupervision is helping. Growth of the field will take time. (Offering) video-based (supervision) sessions. Increasing face to face supervision instead of remote.
	Supervisee Support	Actions that individuals and/or organizations can take, including the development of resources, that will help supervisees access supervision	11%	Local organizations to help connect students with supervisors. Increase in supervisor support forums/SIG/active communities focused on connecting and up skilling supervisors.
	Expanding Fieldwork Opportunities	Creating more fieldwork opportunities (except for university-delivered clinical placements/practicums/fieldwork that should be coded under “University placements”)	9%	An “internship” like structure where supervision must occur in a number of different behavior-analytic fields. Organizations with a supervision model built in.
	Support—Governing Bodies	Establishing a national or local body that is relevant to the work (including supervision) of behavior analysts	9%	Guidelines (for supervision) from the (Australian behavior analysis) association A local . . . board and regulatory body.
	Modify Requirements	Modifying supervision requirements in ways that would assist supervisees to achieve certification	6%	My BCaBA qualification is currently on inactive status as I am unable to meet supervision hours. However, I do receive 1 hour of group supervision and have a needs-based access to individual supervision from 3 BCBAs, however I cannot count this. Particularly within Australia, more flexibility and achievable frequency and total hours of supervision required for BCaBA. This could be remedied by engaging in more CEUs vs supervision hours.

## Discussion

The overarching purpose of the current study was to examine the supervision practices of BCBA certificants in Australia, the perceived challenges related to the provision of supervision, and possible solutions to perceived challenges. We found that most respondents were currently providing supervision or planned to provide supervision in the next 3 years. There was an observed positive correlation between the years of experience of the supervisor and the number of trainees they were supervising, suggesting more experienced BCBA certificants may have more interest in supervising or more capacity to deliver supervision. Respondents reported using a variety of supervisory practices to meet the demand for supervision, including providing “on the job” and external supervision using both in-person and telesupervision models. However, several barriers related to the provision of high-quality supervision were identified, including a lack of time and resources on part of the supervisor, and geographical location of both supervisors and trainees. The most commonly identified barrier for people seeking supervision (e.g., trainees) was a lack of qualified supervisors in Australia. Survey respondents suggested that access to support for supervisors in the form of training, resources, and networking opportunities, as well as the provision of supervised experiences via university run clinical placements, practicums, or fieldwork, might address identified barriers. In what follows, we provide a discussion of these findings and provide recommendations for addressing potential barriers to delivering supervision to trainees in Australia.

The first purpose of the current study was to survey BACB certificants in Australia to determine their existing and future capacity to deliver supervision to trainees. Nearly three quarters of respondents indicated that they were providing supervision, with the same proportion indicating they had done so in the past. It is encouraging that 90% of respondents indicated that they intended to offer supervision in the ensuing 3 years, and the mean number of intended supervisees was also anticipated to increase over this time period. Although it is possible that the high proportions reflect respondent bias to the survey (e.g., those who currently supervise are more likely to complete a survey about supervision) it is nevertheless apparent that a substantial proportion of the relatively small overall population of behavior practitioners in Australia are committed to supporting the training of future practitioners.

A second purpose of the study was to identify and describe the models that Australian BACB certificants currently use to deliver supervision to trainees, including mode (e.g., in-house versus external; in-person versus telesupervision) and format (e.g., individual versus group supervision). The majority of respondents reported offering only individual supervision, or a mix of individual and group supervision. Telesupervision was as frequently used as in-person supervision to deliver both individual and group supervision, with a small number of respondents who reported delivering only group supervision using telesupervision models. It is noteworthy that data collection occurred during the first wave of COVID-19, which may have influenced the strong support for telesupervision. However, other factors in the Australian context, such as the relatively small number of supervisors and supervisees distributed over a large geographical area, likely also contributed to support for telesupervision. The apparent support for the use of telesupervision can also be viewed as encouraging in that it should serve to reduce a key barrier to the establishment of the profession across a large geographical area, meaning that services are more likely to reach communities seeking support from behavior practitioners.

A third purpose of the current study was to identify the barriers that Australian BACB certificants face when delivering supervision to trainees, and to propose potential solutions to commonly identified barriers. The most commonly identified barrier to delivering supervision was time. Respondents noted that the delivery of supervision was challenging because of caseload sizes for BCBAs. Given the relatively small number of BACB certificants in Australia and the increasing demand for ABA-based services, it is likely that Australian BCBAs are experiencing significant pressure to design and supervise the implementation of client programs. One respondent noted that they were unable to allocate time for supervision due to the amount of time that was required to supervise client programs. Another respondent noted that little time was provided to prepare for and deliver high quality supervision. These findings echo those of Sellers et al. (2019), who surveyed American BCBAs and found that time to implement high-quality supervisory practices was one of the most common barriers to effective supervision. Related to this, other respondents articulated concerns related to their own scope of competence to deliver supervision. This is not surprising, given that the majority of survey respondents reported receiving their professional credential within the past 5 years. In addition, respondents noted that geographic location was a barrier to the delivery of supervision, with BCBAs often tasked with supervising trainees in other parts of the state or country. Indeed, we found that telesupervision was used as often if not more frequently as in-person supervision. This means that relatively inexperienced BCBAs must also be fluent with the use of technology, such as videoconferencing, video modelling, video sharing, and video-based feedback, to deliver supervision, in addition to developing their supervisory skills in the areas of demonstrating new skills, shaping the ethical and professional repertoires of supervisees, and providing meaningful performance feedback.

As the field of applied behavior analysis continues to grow in Australia, it is likely that relatively new BACB certificants will experience more pressure to deliver supervision to trainees, and more trainees will have trouble finding a BCBA with the time and skills to provide supervision. If BCBAs take on too much supervisory responsibility too soon, it may inadvertently jeopardize both the quality of supervision provided to students *and* the quality of clinical programming for clients. If trainees cannot find supervisors or are dissatisfied with their supervised experience, they may work outside their scope of practice or competence, deliver lower quality programs to clients, and/or leave the profession entirely. These challenges may pose a risk to the growth of the profession in Australia because both may directly result in client dissatisfaction with ABA-based programs. However, this challenge is not unique to Australia. In fact, Sellers et al. (2016a) noted that the rapid growth in the number of BCBAs between 2010 and 2015, particularly in North America, raised concerns about the degree to which BCBAs were adequately prepared to deliver supervision to the growing workforce and meet the expectations of state and national boards and licensing bodies. The authors noted that, despite providing clear requirements about eligibility to sit for the professional certification exam, the BACB deferred to supervisors to determine the specific content and strategies used to deliver supervision.

Respondents proposed several potential solutions to address the identified barriers for supervisors and supervisees. Many respondents suggested that more supervisor training and support opportunities would be helpful, with responses indicating not only a desire for locally relevant resources, but also a desire for mentorship for supervisors so that they can continue to improve the support they offer. Respondents also suggested that students having access to clinical practicums, expanding fieldwork opportunities, and creating networks to connect trainees with supervisors would help address existing barriers. Individually and combined, these suggestions indicate a desire for more formal and organized networks of support for supervisors and supervisees. It is noteworthy that 20% of respondents referred to increasing financial support as a potential solution and suggested that this could be achieved through better funding for behavior analytic services. Although not mentioned explicitly, the apparent support for greater networking between supervisors may also present opportunities for practitioners to share their models of supervision with colleagues (including costs) in the interests of developing, sharing, and adopting sustainable models more widely.

Based on the findings of this study, we offer three recommendations for addressing potential barriers to delivering supervision to trainees in Australia. These recommendations represent a synthesis of solutions proposed by survey respondents and recommendations or actions described in previous research. Each recommendation has been contextualized to meet the needs of the Australian behavior analytic community. However, these recommendations may be useful in other parts of the world where behavior analysis is an emerging profession or there are few BCBAs to meet the needs of a growing behavior analytic workforce.

### Recommendation #1

Our first recommendation is that a national supervision taskforce be established to support the growth of supervision capacity in Australia. The taskforce could fall under the auspice of the peak national body—the Association for Behaviour Analysis Australia (ABAA)—and comprise practitioners, representatives of university programs and other relevant organizations, and consumers of behavior analytic services. Possible functions of the taskforce could include (1) developing a strategic plan for growing supervision capacity in Australia; (2) systematically identifying and addressing barriers to supervision; and (3) liaising with other peak bodies (e.g., Occupational Therapy Australia, Australian Psychological Society, Speech Pathology Australia) with the view to learning from, and contributing to, the existing body of knowledge and practices for supporting the supervision of allied health practitioners in the Australian context.

### Recommendation #2

Our second recommendation is that a supervisor registry be established in Australia to make it easier for supervisors to advertise opportunities and for trainees to find supervisors. The register could be developed in conjunction with the development of a national registry of BCBAs, that is currently underway as part of the development of an Australian system of professional self-regulation. Although an international register already exists (BACB, 2020a) the findings of this survey indicate that current trainees would value the sharing of more specific information, such as the supervisor’s availability (currently offering/at capacity/internal or external to their own organization), format (individual vs. group), mode (in person, telesupervision, or both), cost for supervisors and their expertise in different applications of ABA (e.g., early intensive behavioral intervention, positive behavior support, risk assessment, acceptance and commitment therapy). Such a register would not only support supervisors and supervisees to connect, it would also provide the proposed national supervision taskforce with regularly updated data on supervision demand, capacity, and characteristics to inform planning and advocacy.

### Recommendation #3

Our third recommendation is that relevant universities and peak bodies work together to establish a support network for supervisors in Australia. The network would provide opportunities for more experienced supervisors to assist in developing training and professional development opportunities for new BCBAs, and to provide mentorship in the area of supervision to new BCBAs. This network could (1) develop telesupervision guidelines that consider Australian regulatory requirements and privacy rules; (2) develop resources to support the practical aspects of supervision (e.g., consent forms for external supervision, tracking forms) to reduce time and support barriers that inhibit or reduce behavior analysts’ capacity to supervise; and (3) create a community of practice to share ideas, and generate local solutions to challenges.

### Strengths and Implications

This study was the first to systematically explore the supervision practices of BACB certificants in Australia. Given the recent establishment of two verified university course sequences in Australia, it is anticipated that the demand for supervision will continue to grow. The current study extends the findings of Leif et al. (2020), who found that the most common barriers ABA practitioners in Australia face are access to training, supervision, and professional development opportunities. In the current study, specific barriers to delivering and accessing supervision in Australia were identified, as well as potential solutions to barriers. These findings have important implications in the Australian context. First, these findings might be used to inform the development of recommendations and strategies for increasing access to high-quality and cost-effective supervision in Australia as the profession continues to grow. Second, the results of this study might be used to inform the development of a national model of self-regulation for behavior analysts in Australia. For example, clear evidence for the demand for supervision, coupled with evidence for constraints in supervision supply—at least in the short term while the profession becomes established—may give weight to proposals to explore models in which supervision could be provided by professionals from across relevant disciplines (e.g., psychology) who are recognized within an Australian professional regulatory framework. Third, these findings might be used to inform the design of a training course for Australian supervisors, similar to the BACBs 8-hour supervisor training course requirement (BACB, 2018), that builds local supervisory competence and prepare Australian supervisors to address barriers to effective supervision in the Australian context.

### Limitations and Future Directions

The findings of this study, although providing the first insight into supervision in the Australian context, is not without its limitations. First, with a geographical focus on Australia only, there is naturally some limitations to the broader relevance of the findings internationally. However, we suggest that this study in fact provides insights into the opportunities and challenges that come with establishing a workforce, along with the supervision support required for this to occur, and that these findings are thus relevant to the expansion of ABA service provision globally. Second, as noted, there is a risk of sampling bias in the current study, in that it is possible that people who currently provide supervision may be more likely to respond. Furthermore, the response rate is not known, as the invitation to participate was shared via professional networks and social media. Future studies could include a direct approach to behavior analysts: a strategy that should be feasible once national registration in Australia is established.

A third limitation is the difference in timing and reporting of the survey findings. As noted, the survey was completed in 2020, meaning that the findings may not fully represent current practice. Since the time of the survey, the number of BCBAs (masters and doctoral) listed on the Australian certificant registry has grown to 216. However, we suggest the findings are nevertheless valuable in exploring the range of issues facing the profession in relation to supervision, including many of which (e.g., number of supervisors) could not conceivably be resolved in the relative short time frame of 2 years. Furthermore, the findings provide a baseline against which the evolution of supervision capacity and practices in Australia can be compared. It is clear that further studies are warranted to document this evolution, and to identify the most current opportunities and pressing barriers related to the delivery of supervision in Australia.

## Conclusion

The findings of this study indicate that there is a strong commitment among behavior analysts in Australia to providing high quality supervision that meets the needs of the growing workforce and as the basis for high-quality clinical services to the community. Nevertheless, a range of barriers to providing, and accessing, supervision were identified including workforce capacity, time, financial considerations, and support for supervisors. Based on the solutions offered by participants, we put forward three recommendations to help grow and support supervision in Australia including establishing a national supervision taskforce, a supervision registry, and a supervision network with each serving complementary roles ranging from strategy to coordination and in person mentoring and support.

## Appendix 1

### Supervision in Applied Behaviour Analysis in Australia: A National Survey

Thank you for agreeing to complete this survey. Please answer all questions to help us gain a rich and complete understanding of the issues.

#### About you and your workplace?

1. First Name:
2. Surname:

- Email address:

3. What is your certification level:
  - BCaBA
  - BCBA
  - BCBA-D
4. Do you hold any other professional qualifications:
  - Allied health
  - Education
  - Other (please specify)
5. Which best describes your current workplace (tick all that apply):
  - Private practice
  - Not-for profit organisation
  - Education provider (e.g., school)
  - Other (please specify)
6. Please estimate how many people are employed in your workplace?
  - 1
  - 2–5
  - 6–10
  - 11–20
  - 21–50
  - 51–100
  - 101+
7. Which best describes your current employment situation?
  - Owner
  - Employee
8. Which best describes your current work profile?
  - Full-time
  - Part-time
9. What is the postcode of your main place of work?
10. In what year did you become certified as a BCBA/BCaBA?
11. Since first becoming certified, how many equivalent full-time years (i.e., taking into consideration any work breaks) have you worked as a BCBA:
  - Less than 1 year
  - 1–2 years
  - 3–5 years
  - 6–10 years
  - 11+ years

#### Your experience providing supervision to those pursing BCaBA/BCBA certification

12. Do you currently offer supervision to people pursuing BCaBA/BCBA certification?
  - Yes
  - No
13. Have you provided supervision to those pursuing BCaBA/BCBA certification in the past?
  - Yes
  - No
14. How many people pursing BCaBA/BCBA certification have you supervised previously?
15. How many people pursing BCaBA/BCBA certification are you currently supervising?
16. Do you offer in-person supervision to those pursuing BCaBA/BCBA certification?
  - Yes
  - No
17. If yes, what models do you offer (tick all that apply):
  - 1:1 supervision
  - Group supervision
18. Do you offer tele-supervision to those pursuing BCaBA/BCBA certification?
  - Yes
  - No
19. If yes, what models do you offer (tick all that apply):
  - 1:1 supervision
  - Group supervision
20. If you offer both in-person and tele-supervision, please estimate the proportion of supervision time that is spent in-person (e.g. 50%)
21. If you offer tele-supervision, what is the main platform you use (e.g., phone, skype, zoom)
22. Are the people you are supervising (tick all that apply):
  - Within your organisation
  - External to your organisation
23. How do people pay for your supervision (tick all that apply):
  - They receive my supervision as part of their employment: They do not pay an additional fee
  - They receive my supervision as part of their employment: They do pay an additional fee
  - They pay my organisation for their supervision
  - They pay me directly for their supervision
24. If you charge a fee for providing supervision, how much do you charge per hour?
25. Do you anticipate your fee going up in the next 24 months?
  - Yes
  - No
  - If yes, what do you anticipate your new fee will be in 24 months’ time (per hour)?
26. If yes, what do you anticipate your new fee will be in 24 months time (per hour)?

#### Thinking to the future

27. Do you plan to provide supervision to those pursuing BCaBA/BCBA certification in the next 3 years?
  - Yes
  - No
28. How many people do you think you might have capacity to supervise in:
  - 2020
  - 2021
  - 2022
29. If you are not planning to supervise those pursuing BCaBA/BCBA certification in the next 3 years, what are the main reasons why? (tick all that apply):
  - Not enough time
  - Not financially viable
  - Do not know the process for getting accredited to supervise
  - Lack of training on how to supervise
  - Lack of resources/tools to support the supervision process
  - Other (please specify)
30. What, if any, do you think are the current barriers for supervisors in Australia?
31. What, if any, do you think are the current barriers for those seeking supervision in Australia?
32. What strategies could help address these barriers?
33. Please share your thoughts and impressions on the following statements:

**Table:**

	Strongly Disagree	Disagree	Neutral	Agree	Strongly Agree
There are currently enough supervisors in Australia to meet student demand					
In the next 3 years, there will be enough supervisors in Australia to meet future demand					
I have the knowledge needed to provide effective supervision (irrespective of whether currently supervising)					
I have the confidence to provide supervision (irrespective of whether currently supervising)					

34. Please share any final thoughts you would like to about the issues raised in this survey, or any other matter your feel is relevant to this research.

We would like to complete this survey annually in Australia as the profession grows, to ensure the two universities can respond to the needs of the community.

35. Are you willing for us to contact you via email for future surveys?
  - Yes
  - No

Thank you very much for the time you have taken to complete this survey and for all the information and insights you have provided. We will forward a summary report once the data have been analysed. Please do not hesitate to let us know if you have any questions in the meantime.
